# Tolerance of Inactivated SARS-CoV-2 Vaccine for People Living with HIV: A Real-World Evidence Analysis from a Retrospective Cohort Study

**DOI:** 10.1007/s44197-025-00452-4

**Published:** 2025-08-14

**Authors:** Qian He, Tao Zhou, Ying Feng, Yuefei Li, Zhen Ni, Ning Zhang, Jing Chen, Mingjian Ni, Shi Zhao, Kai Wang

**Affiliations:** 1 School of Public Health, Xinjiang Medical University, Urumqi, 830017 China; 2 Urumqi Maternal and Child Health Hospital, Urumqi, 830000 China; 3 Xinjiang Uygur Autonomous Region Centre for Disease Control and Prevention, Urumqi, 830002 China; 4 Xinjiang Uygur Autonomous Region Academy of Preventive Medicine, Urumqi, 830002 China; 5 Xinjiang Key Laboratory for AIDS Prevention and Control Research, Urumqi, 830002 China; 6 Yining Second People’s Hospital, Yining, 835000 China; 7 School of Public Health, Tianjin Medical University, Tianjin, 300070 China; 8 Tianjin Key Laboratory of Environment, Nutrition and Public Health, Tianjin Medical University, Tianjin, 300070 China; 9 MoE Key Laboratory of Prevention and Control of Major Diseases in the Population, Tianjin, 300070 China; 10 Department of Medical Engineering and Technology, Xinjiang Medical University, Urumqi, 830017 China

**Keywords:** People living with HIV, SARS-CoV-2, Vaccine safety, Viral rebound, Tolerance

## Abstract

**Background:**

The tolerance of inactivated SARS-CoV-2 vaccines in people living with HIV (PLWH) remains unclear. We aimed to evaluate the tolerance of inactivated SARS-CoV-2 vaccines in PLWH.

**Methods:**

This retrospective cohort study recruited 3327 PLWH for questionnaires and laboratory testing. Subjects were screened to ensure they were receiving antiretroviral therapy for PLWH without SARS-CoV-2 infection. Poisson regression analyses were conducted to assess the association between vaccination and HIV viral rebound, estimating absolute risk difference and relative risk (RR).

**Results:**

A total of 724 PLWH without SARS-CoV-2 infection participated in this study. No significant increase in HIV viral rebound risk was observed after vaccination in the 1/2-dose, 3-dose, and 4-dose groups compared to the 0-dose group. The RRs for the 1/2-dose, 3-dose, and 4-dose groups were 1.22 (95% confidence interval [CI]: 0.55, 2.72), 0.90 (95% CI: 0.48, 1.69), and 1.01 (95% CI: 0.35, 2.89), respectively. Similar results were observed across subgroups. Post-vaccination adverse reactions were minimal, occurring in 2.16% of cases, mostly fatigue and muscle soreness.

**Conclusion:**

Our study suggests that inactivated SARS-CoV-2 vaccines do not adversely affect the risk of HIV viral rebound and were well-tolerated in PLWH.

**Supplementary Information:**

The online version contains supplementary material available at 10.1007/s44197-025-00452-4.

## Introduction

After HIV infection, people living with HIV (PLWH) have compromised immune system and are more likely to be comorbid with other diseases. PLWH with SARS-CoV-2 infection may result in adverse outcomes, particularly those with advanced AIDS, low CD4 levels, or non-suppressed viral load (VL), where rates of hospitalization, severe disease, and mortality may be higher [1–3]. Some studies indicate that antiretroviral therapy (ART) may provide a protective effect [4, 5].

In response to the severe global SARS-CoV-2 pandemic, rapid vaccine development and vaccination represent cost-effective preventive measures for epidemic control. Immunocompromised patients with persistent SARS-CoV-2 infection may develop new SARS-CoV-2 variants [6], and to avoid widespread transmission of COVID-19 as well as mutation in immunocompromised patients, priority should be given to COVID-19 vaccination for this population to mitigate persistent SARS-CoV-2 infection and to avoid adverse outcomes. The World Health Organization (WHO) recommends that PLWH be prioritized for SARS-CoV-2 vaccination [7]. Similarly, the US Centers for Disease Control and Prevention (CDC) recommends that PLWH who are eligible should get SARS-CoV-2 vaccines regardless of viral load or CD4 T lymphocyte cell count, and SARS-CoV-2 vaccines should be given to prevent persistent SARS-CoV-2 infections [8]. In the face of the dual challenges posed by HIV and SARS-CoV-2, vaccination enables PLWH to acquire protective immunity and effective prevention [9]. Inactivated vaccines—characterized by non-replicating properties [10]—are prioritized for PLWH due to lower adverse event risks in immunocompromised populations. A meta-analysis of multiple small-scale studies has indicated that, compared to mRNA vaccines, inactivated vaccines exhibit a lower incidence of adverse events in PLWH, demonstrating good immunogenicity and efficacy [11]. Additionally, an editorial published in The Lancet HIV advocated for prioritizing the SARS-CoV-2 vaccine for PLWH, emphasizing the importance of protecting clinically vulnerable populations and saving lives [12].

Although inactivated SARS-CoV-2 vaccine safety and efficacy have been demonstrated in the general population [13, 14], relevant studies in PLWH remain limited by small sample sizes. Several small-scale studies demonstrate the safety and efficacy of the inactivated SARS-CoV-2 vaccine in PLWH. However, the safety of large-scale studies in PLWH populations receiving ART remains uncertain. The current evidence was limited and incomplete, and further research in large samples of PLWH populations was needed [15]. Second, few studies have explored the safety of the vaccine from the perspective of treated PLWH without SARS-CoV-2 infection, aiming to exclude the effects of SARS-CoV-2 infection on PLWH and avoid confusing active and passive immunity-induced symptoms related to post-vaccination as well as vaccine safety. It is worth mentioning that concerns about vaccine safety are an important influence on vaccination hesitancy [16, 17]; therefore, understanding the safety of the inactivated SARS-CoV-2 vaccine in PLWH may help to improve vaccine coverage and reduce the risk of adverse prognosis.

In this study, we aimed to investigate the individual tolerability and safety of inactivated SARS-CoV-2 vaccination in PLWH and the vaccination rate of the vaccine in the PLWH population. We evaluated the tolerance of the inactivated SARS-CoV-2 vaccine and post-vaccination related symptoms based on PLWH not infected with SARS-CoV-2 in an HIV co-infection cohort in Yining, Xinjiang.

## Patients and Methods

### Study Design and Participants

We performed a retrospective cohort study in Yining city, Xinjiang, China. Participants were recruited from a cohort of HIV-infected patients through local antiviral treatment institutions, with a total of study subjects enrolled from March 2 to May 30, 2023. During this period, a round of follow-up HIV VL testing was conducted. SARS-CoV-2 vaccinations for participants were uniformly administered by the local government, and the number of vaccine doses received varied according to individual health conditions. This included individuals who had not been vaccinated as well as those who received 1 to 4 doses. The inclusion criteria for study subjects were as follows: (1) positive HIV antibody status; (2) long-term residency in Yining City; (3) currently receiving ART; and (4) at least one VL test recorded before and after SARS-CoV-2 vaccination. The exclusion criterion was individuals who had been infected with SARS-CoV-2. We followed the Strengthening the Reporting of Observational Studies in Epidemiology (STROBE) reporting guidelines throughout the investigation.

### Data Collection

A face-to-face electronic questionnaire survey was conducted with all research subjects. It was conducted in the form of oral narration by the patient, consultation, and recording by the doctor. The demographic information collected included sex, age, highest education level, marital status, height, weight, and self-reported smoking status. Additionally, HIV medical history and relevant clinical data were gathered, encompassing the route of HIV transmission, baseline HIV VL test results, post-vaccination HIV VL test results, ART status, duration of ART, and ART classification. Information regarding SARS-CoV-2 infection status and SARS-CoV-2 vaccination was also documented, including vaccination dose and date, vaccine type, and any symptoms or signs experienced after vaccination. Each study subject was assigned a unique code that links the questionnaire responses to their laboratory test results. Post-vaccination adverse events manifested as the development of ten signs and symptoms, Reactions after vaccination including: (1) fever (37.3 degrees centigrade and above), (2) Dry cough, (3) Lack of energy, (4) sore throat, (5) muscle pain, (6) decreased or loss of sense of smell, (7) hypogeusia or loss of taste, (8) diarrhoea, (9) nasal congestion, runny nose, (10) eye symptoms (conjunctivitis). The COVID-19 vaccines administered to the study population included only those vaccines that have been approved in China. VL testing was conducted during the study to collect the most recent post-vaccination HIV VL results. The inactivated SARS-CoV-2 vaccine received by the study subjects was exclusively that approved in China. Samples for VL testing were collected during the research investigation to obtain the latest post-vaccination HIV VL results.

### Outcomes

Eligible study participants who received the inactivated SARS-CoV-2 vaccine and those who did not were enrolled in the exposed and control groups, respectively. In the exposed group, subjects were further categorized based on the number of vaccine doses received into 1/2-dose group (those who received either 1 or 2 doses of the vaccine were less likely to be combined into a single group), 3-dose group, and 4-dose group. The primary outcome of this study was HIV viral rebound before and after inactivated SARS-CoV-2 vaccination, defined as the transition from a suppressed VL (< 1000 copies per mL) to an unsuppressed VL (≥ 1000 copies per mL) [18, 19]. HIV VLs were compared both before and after vaccination, as well as between vaccination doses. Secondary outcomes were post-vaccination adverse events, which encompassed signs and symptoms following SARS-CoV-2 inactivated vaccination.

### Laboratory HIV Viral Load Test

Subjects were recruited based on HIV follow-up efforts and underwent HIV viral load testing. After obtaining informed consent from the study subjects, 5 mL of venous blood was collected using EDTA anticoagulation tubes, and the blood samples at each treatment site were kept refrigerated at 2–8 °C and sent to the Second People’s Hospital of Yining City within 24 h for VL testing. HIV viral load was measured using the COBAS AmpliPrep/COBAS TaqMan HIV-1 Test V.2.0 kit and the Cobas AmpliPrep /Cobas TaqMan48 fully automated analysis system; the lower limit of VL detection was 20 copies per ml.

### Statistical Analyses

In this study, we evaluated the changes in HIV VL following vaccination. Descriptive statistics were initially computed to summarize the demographic and clinical characteristics of the participants by vaccine status. Poisson regression and paired t-tests were used to analyze the association between the inactivated SARS-CoV-2 vaccine and HIV VL according to the attributes of VL count data and measurement data. In categorical data, two measures of effect size were adopted: the absolute risk difference (ARD) and the relative risk (RR) value. ARD refers to the difference in overall risk of harm in the HIV VL values between four different vaccine dose groups. RR showing differences in the incidence of adverse outcomes in HIV VL values between four different vaccine dose groups. In the multivariate analyses, RR was adjusted for sex, age, highest education level, marital status, route of HIV transmission, BMI, ART classification, self-reported smoking status, and preexisting conditions. In measurement data, two measures of effect size were adopted: the absolute unit difference and percentage change. The absolute unit difference refers to the difference in overall risk of harm in the HIV VL value between four different vaccine dose groups. The RR shows differences in the incidence of adverse outcomes in HIV VL value between four different vaccine dose groups. In addition, subgroup analyses were conducted to further understand the differences in vaccine responses by sex, age groups, and illness histories. All statistical tests were two-sided, and a *P*-value < 0.05 was considered statistically significant.

The R statistical software (version 4.0.4) was used for all statistical analyses.

## Results

**Fig. 1:**
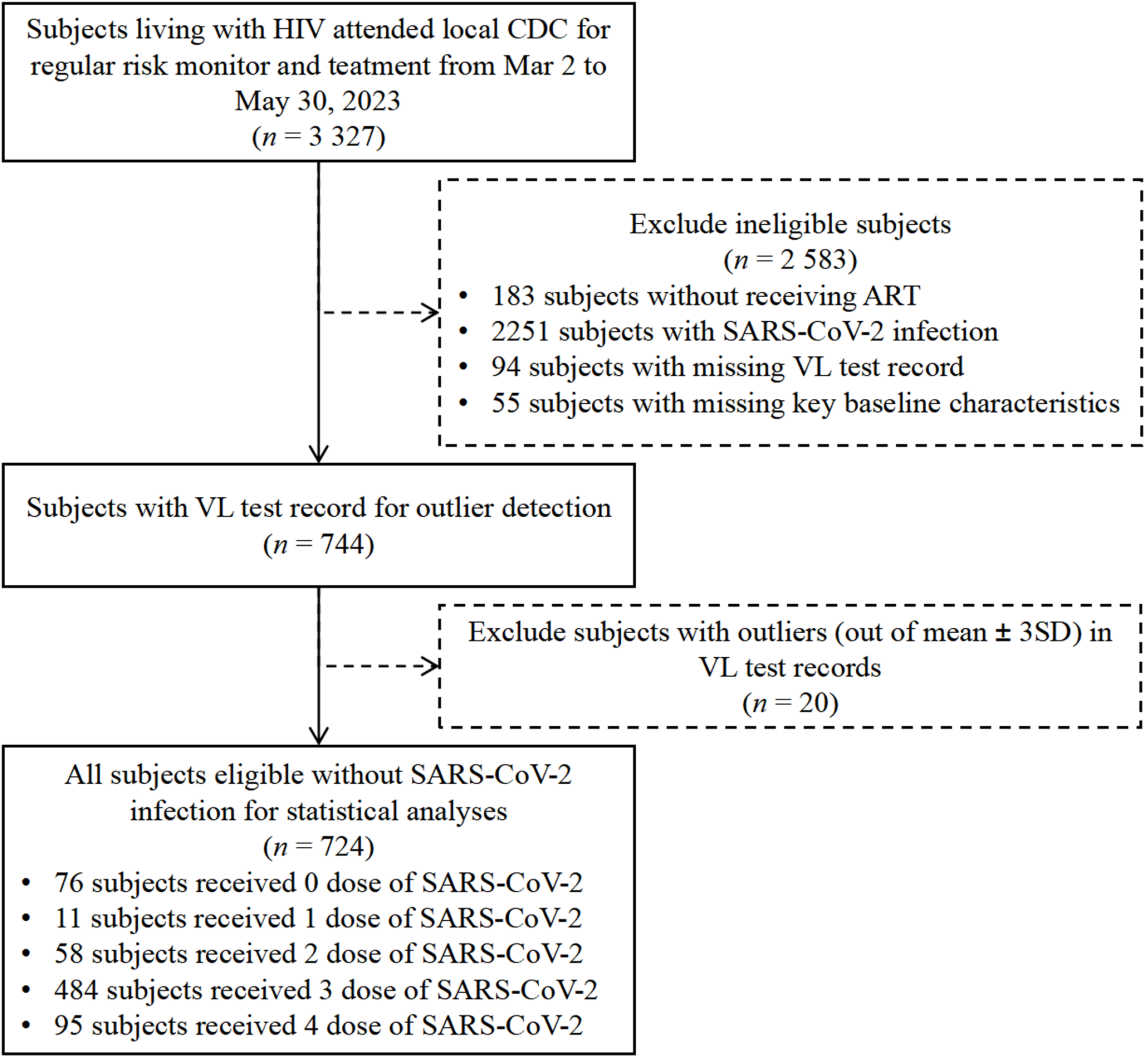
Flow diagram of subject selection

A total of 3327 PLWH were recruited in this study, of whom 183 subjects were excluded because they were not receiving ART, 2251 subjects were excluded because they self-reported with SARS-CoV-2 infection, and 168 subjects were excluded because of missing values or outliers in the baseline characteristics. According to whether they were inactivated SARS-CoV-2 vaccines and the number of doses received, 724 subjects ultimately were divided into four groups: the 0-dose group (76 subjects, 10.62%), the 1/2-dose group (69 subjects, 9.52%), the 3-dose group (484 subjects, 66.76%), and the 4-dose group (95 subjects, 13.10%), Inactivated SARS-CoV-2 vaccines coverage for PLWH in this study was 89.4%. A detailed flow chart is shown in Fig. 1.

Table 1 shows the demographic and clinical characteristics of the groups with different doses of the SARS-CoV-2 vaccine in PLWH without SARS-CoV-2 infection. 724 subjects were observed, most of whom were 25–50 years (434/724, 59.9%), the median age was 46.0 years (IQR: [39.0, 52.0]), and male (451/724, 62.3%); 285 (39.4%) were primary school or below and 274 (37.8%) were secondary school; the major route of HIV transmission was heterosexual transmission (451/724, 62.3%), followed by injecting drug use (180/724, 24.9%), with a median duration of HIV infection of 10.0 years (IQR: [5.5, 13.5]); and more PLWH without pre-existing condition (76.2%); the majority of the participants had an undetectable HIV VL (< 20 copies per mL) (452/724, 62.4%); most of PLWH were married or living with partners (336/724, 46.4%); never smokers and current smoker were equally divided. For each dosage group, 47 subjects (61.8%) HIV VL was undetectable in the 0-dose group, 32 (46.4%) in the 1/2-dose group, 313 (64.7%) in the 3-dose group, 60 (63.2%) in the 4-dose group; 36.8% of PLWH with pre-existing condition in the 0-dose group and was higher than other groups; the median duration of ART was 8.2 years [4.7, 11.2] in the 3-dose group and was slightly higher than other groups.

**Table 1:** Demographic and clinical characteristics of the eligible cohorts of PLWH without SARS-CoV-2 infection

Baseline characteristics	Vaccine status before ejaculation (column %)				
	Total subjects	0-dose vaccine	1/2-dose vaccine	3-dose vaccine	4-dose vaccine
Total sample size	724(100%)	76 (100%)	69 (100%)	484 (100%)	95 (100%)
**Sociodemographic characteristics**					
*Age*, yr, *n (%)*					
<25	40 (5.5%)	7 (9.2%)	11 (15.9%)	20 (4.1%)	2 (2.1%)
25–50	434 (59.9%)	50 (65.8%)	42 (60.9%)	287 (59.3%)	55 (57.9%)
50–65	221 (30.5%)	16 (21.1%)	9 (13.0%)	160 (33.1%)	36 (37.9%)
≥65	29 (4.0%)	3 (3.9%)	7 (10.1%)	17 (3.5%)	2 (2.1%)
*Median age*,* yr [IQR]*	46.0 [39.0, 52.0]	45.0 [39.0, 49.3]	42.0 [32.0, 49.0]	46.0 [39.8, 52.0]	46.0 [40.5, 52.0]
*Sex*,* n (%)*					
Male	451 (62.3%)	51 (63.8%)	45 (65.2%)	294 (60.7%)	62 (65.3%)
Female	273 (37.7%)	26 (34.2%)	24 (34.8%)	190 (39.3%)	33 (34.7%)
*Highest education level*,* n (%)*					
Primary school or below	285 (39.4%)	30 (39.0%)	40 (58.0%)	191 (39.5%)	24 (25.3%)
Secondary school	274 (37.8%)	31 (40.8%)	24 (34.8%)	177 (36.6%)	42 (44.2%)
Associated degree or above	165 (22.8%)	15 (19.7%)	5 (7.2%)	116 (24.0%)	29 (30.5%)
*Median duration of formal education*,* yr [IQR]*	9.0 [6.0, 9.0]	9.0 [6.0, 9.0]	6.0 [6.0, 9.0]	9.0 [6.0, 9.0]	9.0 [7.5, 12.0]
*Marital status*,* n (%)*					
Single	188 (26.0%)	26 (34.2%)	30 (43.5%)	108 (22.3%)	24 (25.3%)
Married or living with partners	336 (46.4%)	30 (39.5%)	20 (29.0%)	241 (49.8%)	45 (47.4%)
Divorced or widowed	176 (24.3%)	17 (22.4%)	16 (23.2%)	120 (24.8%)	23 (24.2%)
Unknown	24 (3.3%)	3 (3.9%)	3 (4.3%)	15 (3.1%)	3 (3.2%)
*Self-reported smoking status*,* n (%)*					
never smoker	360 (49.7%)	35 (46.1%)	32 (46.4%)	249 (51.4%)	44 (46.3%)
current smoker or former smoker (quit before 6 months)	364 (50.3%)	41 (53.9%)	37 (53.6%)	235 (48.6%)	51 (53.7%)
*Route of HIV transmission*,* n (%)*					
Injecting drug use	180 (24.9%)	31 (40.8%)	22 (31.9%)	109 (22.5%)	18 (18.9%)
Heterosexual transmission	451 (62.3%)	33 (43.4%)	30 (43.5%)	325 (67.1%)	63 (66.3%)
Homosexual transmission	28 (3.9%)	1 (1.3%)	3 (4.3%)	20 (4.1%)	4 (4.2%)
Mother-to-foetus transmission	30 (4.1%)	4 (5.3%)	10 (14.5%)	14 (2.9%)	2 (2.1%)
Others	35 (4.8%)	7 (9.2%)	4 (5.8%)	16 (3.3%)	8 (8.4%)
*Body mass index group*,* n (%)*					
underweight: BMI < 18.5 kg/m^2^	40 (5.5%)	8 (10.5%)	5 (7.2%)	22 (4.5%)	5 (5.3%)
normal weight: BMI 18.5–24.9 kg/m^2^	424 (58.6%)	47 (61.8%)	39 (56.5%)	277 (57.2%)	61 (64.2%)
overweight: BMI 25–30 kg/m^2^	202 (27.9%)	15 (19.7%)	18 (26.1%)	145 (30.0%)	24 (25.3%)
obesity: BMI > 30 kg/m^2^	58 (8.0%)	6 (7.9%)	7 (10.1%)	40 (8.3%)	5 (5.3%)
*Median BMI*,* kg/m*^*2*^ *[IQR]*	23.9 [21.3, 26.3]	22.9 [20.6, 25.4]	23.4 [21.1, 26.5]	24.1 [21.5, 26.5]	24.1 [22.1, 25.5]
*Pre-existing conditions*,* n (%)*					
0	553 (76.3%)	49 (63.6%)	53 (76.8%)	370 (76.4%)	81 (85.3%)
≥1	172 (23.7%)	28 (36.4%)	16 (23.2%)	114 (23.6%)	14 (14.7%)
*Median duration of HIV infection*,* yr [IQR]*	10.0 [5.5, 13.5]	9.8 [5.4, 14.3]	10.4 [5.4, 13.3]	9.9 [5.5, 13.3]	9.8 [5.6, 13.5]
**Baseline laboratory test results**					
*Plasma viral load*,* copies per mL*					
< 20	452 (62.4%)	47 (61.8%)	32 (46.4%)	313 (64.7%)	60 (63.2%)
20–200	89 (12.3%)	7 (9.2%)	10 (14.5%)	57 (11.8%)	15 (15.8%)
200–400	28 (3.9%)	3 (3.9%)	4 (5.8%)	18 (3.7%)	3 (3.2%)
400–1000	38 (5.2%)	5 (6.6%)	5 (7.2%)	25 (5.2%)	3 (3.2%)
≥1000	117 (16.2%)	14 (18.4%)	18 (26.1%)	71 (14.7%)	14 (14.7%)
*Median viral load*,* copies per mL [IQR]*	0.0 [0.0, 211.5]	0.0 [0.0, 375.5]	46.0 [0.0, 1440.0]	0.0 [0.0, 138.0]	0.0 [0.0, 57.0]
**Baseline HIV theraputic treatment**					
*Antiretroviral therapy classification*					
WHO recommended first-line ART regimens	474 (65.5%)	43 (56.6%)	35 (50.7%)	327 (67.6%)	69 (72.6%)
WHO recommended second-line ART regimens	250 (34.5%)	33 (43.4%)	34 (49.3%)	157 (32.4%)	26 (27.4%)
*Duration of ART*, *yr [IQR]*	8.2 [4.7, 11.3]	8.1 [4.6, 11.3]	7.9 [4.8, 10.7]	8.2 [4.7, 11.2]	8.0 [4.6, 11.3]

We monitored the HIV VL values before and after COVID-19 inactivated vaccination and classified the virological status of PLWH as VL suppressed (VL < 1000 copies per mL) and viral rebound (VL ≥ 1000 copies per mL) based on this VL. Analysis of the virological status of PLWH showed no significant difference in the virological status before and after vaccination (*P* > 0.05), nor between the different doses of vaccination (*P* > 0.05), and the results of this study showed no evidence that vaccination with the inactivated SARS-CoV-2 vaccine induced HIV viral rebound (Table 2). A slightly higher ARD in the 1/2-dose group (9.12%) than in the 0-dose group, 3-dose group (-5.20%), and 4-dose group (-7.89%) were slightly lower than 0-dose group. In multivariate models, the adjusted RR for PLWH viral rebound was 1.22 (95% CI: 0.55, 2.72) in the 1/2-dose group, 0.90 (95% CI: 0.48, 1.69) in the 3-dose group, and 1.01 (95% CI: 0.35, 2.89) in the 4-dose group. In subgroup analysis, the results showed a similar trend of the ARD that is in the 1/2-dose group was slightly higher than in the 0-dose group, 3-dose group and 4-dose group were slightly lower than in the 0-dose group, which were observed when analyzing in the subgroups male, aged ≥ 40 years, without any pre-existing condition, with any pre-existing condition (≥ 1); However, a contrary trend was observed in females, aged < 40years, where the ARD between 1/2-dose group, 3-dose group, and 4-doses group were higher than that of the 0-dose group, but the adjusted were not significant, suggesting that there is no risk of inducing viral load unsuppression with any dose of vaccine.

**Table 2:** Effects of 0, 1/ 2, 3, and 4 doses of vaccine on viral rebound (VL ≥ 1000 copies per mL) outcomes of PLWH without SARS-CoV-2 infection (VL < 1000 copies per mL as the control group)

Stratification	Sample size	Event at latest follow-up (%)	ARD	RR (95% CI)	
				crude	adjusted^$^
Overall					
0-dose (ref.)	76	14 (18.42%)	1.00 (ref)	1.00 (ref)	1.00 (ref)
1/2-dose vaccine	69	19 (27.54%)	9.12%	1.50 (0.75, 2.98)	1.22 (0.55, 2.72)
3-dose vaccine	484	64 (13.22%)	-5.20%	0.72 (0.40, 1.28)	0.90 (0.48, 1.69)
4-dose vaccine	95	10 (10.53%)	-7.89%	0.57 (0.25, 1.27)	1.01 (0.35, 2.89)
among female subjects					
0-dose (ref.)	26	3 (11.54%)	1.00 (ref)	1.00 (ref)	1.00 (ref)
1/2-dose vaccine	24	5 (20.83%)	9.29%	1.81 (0.43, 7.56)	(not calculated)
3-dose vaccine	190	23 (12.11%)	7.38%	1.05 (0.32, 3.49)	0.63 (0.17, 2.29)
4-dose vaccine	33	1 (12.63%)	1.09%	0.26 (0.03, 2.53)	(not calculated)
among male subjects					
0-dose (ref.)	50	11 (22.00%)	1.00 (ref)	1.00 (ref)	1.00 (ref)
1/2-dose vaccine	45	14 (31.11%)	9.11%	1.41 (0.64, 3.12)	1.50 (0.54, 4.18)
3-dose vaccine	294	41 (13.95%)	-8.05%	0.63 (0.33, 1.23)	0.82 (0.39, 1.75)
4-dose vaccine	62	9 (14.52%)	-7.48%	0.66 (0.27, 1.59)	1.83 (0.50, 6.75)
among subjects with age < 40 yr					
0-dose (ref.)	22	3 (13.64%)	1.00 (ref)	1.00 (ref)	1.00 (ref)
1/2-dose vaccine	26	10 (38.46%)	24.82%	2.82 (0.78, 10.25)	5.24 (0.63, 43.73)
3-dose vaccine	121	27 (22.31%)	8.67%	1.64 (0.50, 5.39)	2.41 (0.63, 9.28)
4-dose vaccine	19	4 (21.05%)	7.41%	1.54 (0.35, 6.90)	(not calculated)
among subjects with age ≥ 40 yr					
0-dose (ref.)	54	11 (20.37%)	1.00 (ref)	1.00 (ref)	1.00 (ref)
1/2-dose vaccine	43	9 (20.93%)	0.56%	1.03 (0.43, 2.48)	0.84 (0.25, 2.77)
3-dose vaccine	363	37 (10.19%)	-10.18%	0.50 (0.26, 0.98)	0.55 (0.26, 1.18)
4-dose vaccine	76	6 (7.89%)	-12.48%	0.39 (0.14, 1.05)	0.31 (0.06, 1.59)
among subjects without Any pre-existing condition					
0-dose (ref.)	48	8 (16.67%)	1.00 (ref)	1.00 (ref)	1.00 (ref)
1/2-dose vaccine	53	15 (28.30%)	11.63%	1.70 (0.72, 4.01)	0.88 (0.33, 2.35)
3-dose vaccine	370	51 (13.78%)	-2.89%	0.83 (0.39, 1.74)	1.01 (0.46, 2.25)
4-dose vaccine	81	9 (11.11%)	-5.56%	0.67 (0.26, 1.73)	0.87 (0.25, 3.08)
among subjects with Any pre-existing condition (≥ 1)					
0-dose (ref.)	28	6 (21.43%)	1.00 (ref)	1.00 (ref)	1.00 (ref)
1/2-dose vaccine	16	4 (25.00%)	3.57%	1.17 (0.33, 4.13)	9.30 (0.04, 2077.25)
3-dose vaccine	114	13 (11.40%)	-10.03%	0.53 (0.20, 1.40)	0.65 (0.18, 2.31)
4-dose vaccine	14	1 (7.14%)	-14.29%	0.33 (0.04, 2.77)	(not calculated)

^**#**^ viral rebound is defined as VL ≥ 1000 copies per mL (Unsuppressed viral load), and VL<1000 copies per mL (Suppressed viral load and undetectable viral load) as the control group. ^**$**^ Effect of 0, 1/ 2, 3, and 4 doses of vaccine on HIV VL in PLWH was estimated from multivariate log-binomial regression models adjusted for covariates including sex, age, highest education level, marital status, route of HIV transmission, BMI, self-reported smoking status, pre-existing conditions, and ART classification administered. Abbreviations: ARD = absolute risk difference; RR = relative risk; CI = confidence interval

**Fig. 2:**
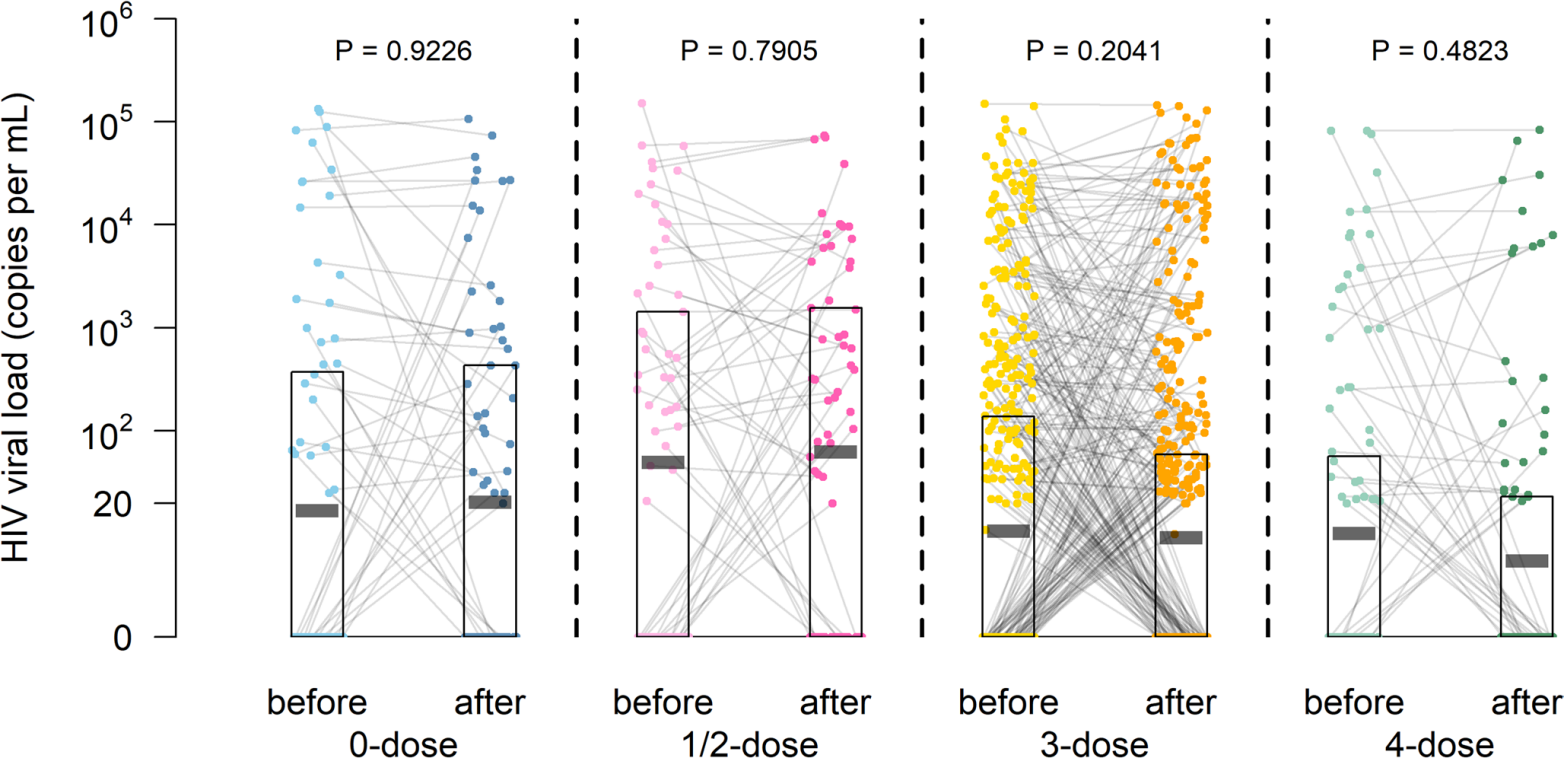
Distribution of the change in VL in PLWH before and after SARS-CoV-2 vaccines. In the plots, the VL values points of the same subjects was linked by the solid grey line. The distributions of VL values points of before and after different doses of vaccination for PLWH were shown by different colours. The black-outlined rectangles and short short translucent black horizontal lines indicated the interquartile ranges and geometric mean, respectively

The signed-rank test was employed to quantitatively compare the VL values of PLWH before and after vaccination as part of the sensitivity analyses. The results indicated that there was no significant difference in VL values before and after vaccination among PLWH who received 0, 1/2, 3, and 4-doses of the vaccine (*P* > 0.05). The 1/2-dose vaccine group exhibited the highest AUD, while the 4-dose vaccine group had the lowest (Table 3). In comparison to the 0-dose group, the VL values of each vaccinated group showed small changes before and after vaccination. Among them, the VL values for the 1/2-dose and 3-dose vaccine groups increased slightly after vaccination, with percentage changes (95% CI) of 6.86% (6.37, 7.34) and 9.90% (9.52, 10.29), respectively. And the VL value decreased slightly after the 4-dose vaccination, with a percentage change (95% CI) of -26.19% (-26.56, -25.82). As also seen in Fig. 2, no significant difference in VL before and after vaccination was observed (*P* > 0.05), where the geometric mean VL before and after vaccination in the 0-dose group was 17.09 vs. 20.56 copies per mL, in the 1/2-dose group: 50.14 vs. 63.61 copies per mL, in the 3-dose group: 10.80 vs. 9.35 copies per mL, and in the 4-dose group: 10.24 vs. 5.54 copies per mL. In subgroup analyses, there were no significant differences in VL values before and after vaccination in PLWH for the 0, 1/2, 3, and 4-dose vaccine groups (*P* > 0.05).

**Table 3:** Demographic and clinical characteristics of the eligible cohorts of PLWH without SARS-CoV-2 infection

Stratification	Level at baseline mean (SD)	Level at latest follow-up mean (SD)	AUD	Percentage change (95% CI)
Overall (*n* = 724)				
0-dose (ref.)	8292.87 (25899.49)	5131.57 (16577.15)	6819.25	0.00% (ref)
1/2-dose vaccine	7128.10 (21713.52)	5140.72 (15064.25)	7139.90	6.86% (6.37, 7.34)
3-dose vaccine	3253.95 (13807.73)	4075.05 (16644.55)	5185.94	9.90% (9.52, 10.29)
4-dose vaccine	3587.14 (14415.68)	2679.31 (11628.00)	3763.41	-26.19% (-26.56, -25.82)
among female subjects (*n =* 273)				
0-dose (ref.)	347.54 (918.83)	986.42 (3027.36)	1013.58	0.00% (ref)
1/2-dose vaccine	13192.29 (34217.07)	5323.96 (15542.64)	13031.08	77.62% (75.16, 80.13)
3-dose vaccine	3432.08 (15771.52)	2639.65 (12737.77)	3280.15	90.01% (94.55, 99.52)
4-dose vaccine	437.24 (1513.76)	189.85 (1073.57)	391.45	-80.72% (-81.25, -80.19)
among male subjects (*n =* 451)				
0-dose (ref.)	12424.44 (31230.82)	7287.04 (20051.97)	9838.20	0.00% (ref)
1/2-dose vaccine	3893.87 (9266.94)	5043.00 (14980.23)	3997.93	20.44% (19.77, 21.11)
3-dose vaccine	3138.82 (12401.64)	5002.69 (18703.81)	6417.56	14.90% (14.45, 15.35)
4-dose vaccine	5263.69 (17629.74)	4004.34 (14234.29)	5558.16	-15.21% (-15.66, -14.76)
among subjects with age < 40 year (*n =* 188)				
0-dose (ref.)	12885.23 (37917.25)	4273.86 (15867.69)	8728.09	0.00% (ref)
1/2-dose vaccine	11324.50 (31561.31)	6322.04 (14911.56)	11005.23	66.18% (64.86, 67.52)
3-dose vaccine	4299.01 (17341.66)	4561.64 (16036.43)	4371.17	96.50% (95.12, 97.92)
4-dose vaccine	3268.21 (7780.46)	2822.79 (6884.90)	4092.16	48.94% (47.32, 50.58)
among subjects with age ≥ 40 year (*n =* 537)				
0-dose (ref.)	6421.91 (19163.49)	5481.00 (16990.52)	6041.57	0.00% (ref)
1/2-dose vaccine	4590.74 (12366.26)	4426.44 (15286.36)	4802.72	-4.30% (-4.85, -3.74)
3-dose vaccine	2905.59 (12417.84)	3912.85 (16860.86)	5457.53	-16.50% (-16.83, -16.16)
4-dose vaccine	3666.87 (15681.09)	2643.43 (12573.01)	3681.22	-45.80% (-46.10, -45.49)
among subjects without Any pre-existing condition (*n* = 553)				
0-dose (ref.)	8309.73 (27671.51)	4253.27 (12775.98)	7860.08	0.00% (ref)
1/2-dose vaccine	8126.26 (24174.77)	5130.32 (14429.88)	8253.98	26.63% (25.90, 27.36)
3-dose vaccine	3356.68 (14411.37)	4429.99 (17537.53)	5247.45	46.74% (46.06, 47.43)
4-dose vaccine	4093.63 (15545.07)	3038.63 (12539.91)	4221.62	1.83% (1.22, 2.43)
among subjects with Any pre-existing condition (≥ 1) (*n* = 172)				
0-dose (ref.)	8263.96 (23029.98)	6637.21 (21805.57)	5034.96	0.00% (ref)
1/2-dose vaccine	3821.69 (9807.78)	5175.19 (17519.16)	3449.50	46.30% (45.02, 47.59)
3-dose vaccine	2920.50 (11686.25)	2923.06 (13344.10)	4986.28	-23.05% (-23.52, -22.57)
4-dose vaccine	656.71 (2172.63)	600.36 (2127.83)	1112.36	-79.93% (-80.36, -79.47)

^**$**^ Effect of 0, 1/ 2, 3, and 4 doses of vaccine on HIV VL in PLWH was estimated from multivariate log-binomial regression models adjusted for covariates including sex, age, highest education level, marital status, route of HIV transmission, BMI, self-reported smoking status, pre-existing conditions, and ART classification administered. Abbreviations: AUD = absolute unit difference; SD = standard deviation; CI = confidence interval

**Fig. 3:**
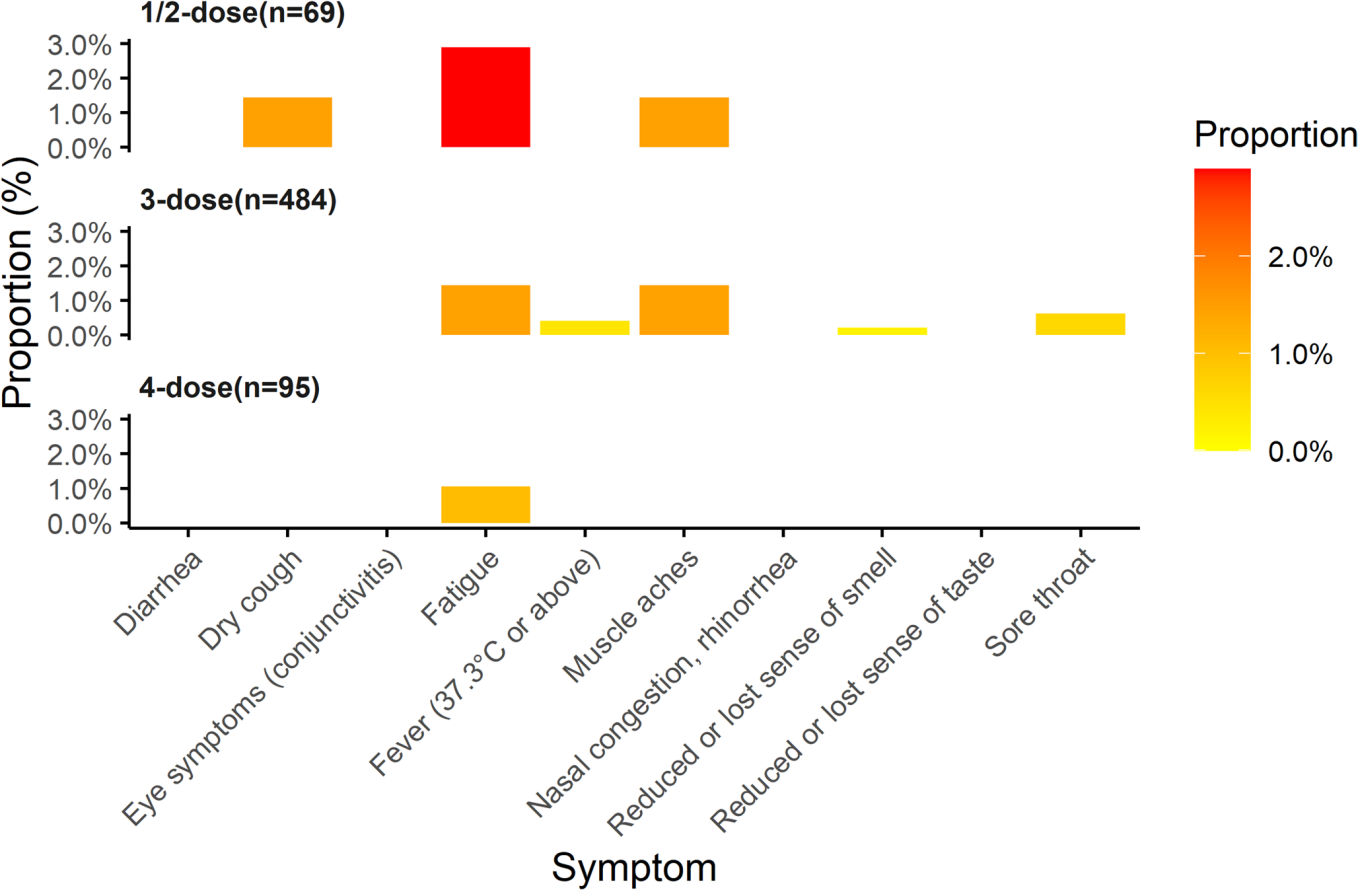
The proportional distribution of symptoms following vaccination with different doses of the vaccine. The colour bar (legend) shows the exact value of the ratio, from 0.0% (yellow) to 3.0% (red)

Meanwhile, we observed adverse reactions after vaccination, which occurred in 14 (2.16%) of 648 vaccinated individuals (Supplementary Table 1). The incidence rate of most symptoms was low, and the adverse reaction rate within each dose group did not exceed 3.0% (Fig. 3). Among these reactions, fatigue and muscle soreness were relatively common.

## Discussion

Limited data exist on inactivated SARS-CoV-2 vaccination responses in PLWH who were without SARS-CoV-2 infection and who were receiving ART. Here, we recruited PLWH who were not infected with SARS-CoV-2 and were receiving ART, and we retrospectively analyzed their doses of inactivated SARS-CoV-2 vaccines, along with their HIV virological status before and after vaccination, and evaluated any adverse reactions. Our study demonstrates that vaccination with the inactivated SARS-CoV-2 vaccine was not associated with an increased risk of viral rebound in PLWH, and there are no safety concerns related to vaccine dose. Furthermore, we observed fewer adverse events following vaccination, with symptoms being mild and of short duration.

In our retrospective cohort study, we found that the COVID-19 vaccination rate among PLWH was 89.5%, which was slightly higher than the results of a nationwide cross-sectional survey in China (88.3%) [20], the results of PISCIS cohort survey (81.4%) [21], and significantly higher than the global vaccination rate for PLWH (56.6%) [22]. This discrepancy can be attributed to a time lag [20], as well as variations in regional vaccination policies, public awareness campaigns, and community acceptance [22]. It is noteworthy that vaccination rates are relatively low among individuals with pre-existing conditions. There was a positive correlation between the number of doses of vaccine and the proportion of individuals with pre-existing conditions. This may be attributed to the increased vaccine anxiety among those with pre-existing conditions, who were concerned about the potential adverse effects of vaccination, which in turn resulted in delays and hesitation in vaccination. This phenomenon was analogous to the findings observed in the early stages of the epidemic [23–25].

Early in the epidemic, PLWH expressed concerns about potential adverse reactions to the SARS-CoV-2 vaccines and their possible negative impact on ART, resulting in relatively low vaccination rates. At the same time, there have been no clear reports of interaction between vaccines and ART [26]. In addition, while HIV suppression is often inadequate to fully mitigate immune activation due to the presence of HIV reservoirs [23, 27]. Several studies have highlighted the role of vaccine-induced T cell activation in HIV replication; however, the impact of vaccination with inactivated vaccines on VL has received significant attention. This study focused on PLWH receiving ART to assess vaccine safety. The results indicated that the risk of viral rebound was slightly higher in those vaccinated with 0 dose than in those vaccinated with 3 or 4 doses, although this difference was not statistically significant. Additionally, the risk of viral rebound was also slightly higher in the group vaccinated with 1/2 doses than in the 0-dose group, but this difference was also not statistically significant. These findings may have been influenced by the timing of VL testing; the majority of individuals receiving 1/2 doses were vaccinated between the second half of 2021 and the first half of 2022. During the early stages of the epidemic, China implemented a dynamic zero-COVID policy, and Xinjiang experienced multiple disruptions due to the COVID-19 epidemic, which may have led to interruptions in ART for some PLWH, resulting in unstable VL control. In subgroup analysis, inactivated SARS-CoV-2 vaccines had the same safety profile after vaccination among PLWH of different genders, ages, and pre-existing conditions, which was consistent with the results of a meta-analysis [28]. Overall, the study concludes that there are no significant risks of viral rebound or vaccine safety issues associated with vaccination. This conclusion aligns with a longitudinal cohort study that found no significant differences in VL before and after vaccination with the inactivated SARS-CoV-2 vaccines, which are deemed safe and effective for PLWH [29]. This was consistent with the findings from several previous small sample studies, which indicated that the safety data for SARS-CoV-2 vaccinations were robust [30, 31]. In comparison to mRNA vaccines, a longitudinal cohort study [32] conducted in Canada also indicated that there is no evidence suggesting that COVID-19 mRNA vaccines induce changes in HIV viremia. However, there are inconsistent results among PLWH who were not receiving ART. One case report highlighted significant increases in VL following SARS-CoV-2 vaccination in HIV-infected individuals who were not on ART [33]. Furthermore, an elite controller with an undetectable VL also experienced an increase in VL post-vaccination. This heterogeneity appears to be more closely associated with the absence of effective ART [31]. Therefore, it was recommended that all PLWH, regardless of their immune status, initiate ART as soon as possible. This approach not only preserves immune function but also minimizes secondary infections associated with HIV. It also mitigates the adverse effects of co-infections, including potential interactions with vaccinations. Furthermore, it is essential to understand the impact of SARS-CoV-2 vaccines on immunocompromised individuals. Ensuring safety and efficacy in these populations is crucial for effective disease surveillance and informing public health policy.

Several limitations are noteworthy. Firstly, as a retrospective observational study, it is inherently susceptible to unmeasured confounding factors and limits causal inference. Specifically, the self-reported history of SARS-CoV-2 infection may introduce recall and reporting bias. Although most of the study period was under extensive government surveillance, with residents receiving frequent nucleic acid or dual-antibody testing, this reliance on self-reporting and the retrospective nature of data collection may still lead to significant misclassification and biased interpretations of our current results. These limitations require mitigation through prospective designs in future research. Secondly, as the vaccines were implemented by the government, the vaccine manufacturers may have varied at different times. It was not possible to control for the distribution of vaccines from different manufacturers, although the majority of vaccines were from the same manufacturer and in the same dose. And all of them were inactivated SARS-CoV-2 vaccines produced in China.

Therefore, it is recommended that all PLWH, regardless of their immune status, initiate ART as soon as possible. This approach not only preserves immune function and minimizes secondary infections associated with HIV, but also mitigates the adverse effects of co-infections, including potential interactions with vaccinations. Furthermore, it is essential to understand the impact of COVID-19 vaccines on immunocompromised individuals. Ensuring safety and efficacy in these populations is crucial for effective disease surveillance and informing public health policy.

## Conclusions

Our study utilized cohort data to investigate the high coverage rate of inactivated SARS-CoV-2 vaccines among PLWH. We found that PLWH receiving ART were administered different doses of the vaccine, and the small, statistically insignificant changes in VL before and after vaccination were negligible. Inactivated SARS-CoV-2 vaccines demonstrated favorable safety profiles among PLWH with varying virological statuses. It is essential to develop relevant public health measures to promote vaccination in this vulnerable population, thereby mitigating or preventing the adverse effects of the SARS-CoV-2 pandemic. Furthermore, ART coverage should be enhanced, and more PLWH should be encouraged to initiate ART as soon as possible to improve resilience against emerging infectious diseases in the future.

## Supplementary Information

Below is the link to the electronic supplementary material.

Supplementary Material 1

## Data Availability

The original database containing confidential patient information cannot be made publicly available. The anonymized data used in this study were available on reasonable request to the corresponding authors.
