# Supplementary material for: Tolerance of Inactivated SARS-CoV-2 Vaccine for People Living with HIV: A Real-World Evidence Analysis from a Retrospective Cohort Study
Source: J Epidemiol Glob Health. 2025 Aug 14;15(1):106. doi: 10.1007/s44197-025-00452-4 (PMC12354443; doi:10.1007/s44197-025-00452-4)
Supplement: Supplementary file 1 — Supplementary Material 1 [file 44197_2025_452_MOESM1_ESM.docx]

**Supplementary materials**

**Supplementary Table 1. Distribution of symptoms after vaccination with different doses of vaccine**

| **Symptoms** | Vaccine status (column %) | | | |
| --- | --- | --- | --- | --- |
|  | Total vaccinees | 1/2-dose vaccine | 3-dose vaccine | 4-dose vaccine |
| **Total sample size** | 648(100%) | 69 (100%) | 484 (100%) | 95 (100%) |
| **Symptoms** | | | | |
| Fever(37.3°C or above) | 2 (0.31%) | 0 (0.0%) | 2 (0.41%) | 0 (0.0%) |
| Dry cough | 1 (0.15%) | 1 (1.45%) | 0 (0.0%) | 0 (0.0%) |
| Fatigue | 10 (1.54%) | 2 (2.90%) | 7 (1.45%) | 1 (1.05%) |
| Sore throat | 3 (0.46%) | 0 (0.0%) | 3 (0.62%) | 0 (0.0%) |
| Muscle aches | 8 (1.23%) | 1 (1.45%) | 7 (1.45%) | 0 (0.0%) |
| Reduced or lost sense of smell | 1 (0.15%) | 0 (0.0%) | 1 (0.21%) | 0 (0.0%) |
| Reduced or lost sense of taste | 0 (0.0%) | 0 (0.0%) | 0 (0.0%) | 0 (0.0%) |
| Diarrhea | 0 (0.0%) | 0 (0.0%) | 0 (0.0%) | 0 (0.0%) |
| Nasal congestion, rhinorrhea | 0 (0.0%) | 0 (0.0%) | 0 (0.0%) | 0 (0.0%) |
| Eye symptoms (conjunctivitis) | 0 (0.0%) | 0 (0.0%) | 0 (0.0%) | 0 (0.0%) |
| Any symptoms | 14 (2.16%) | 2 (2.90%) | 11 (2.27%) | 1 (1.05%) |
